# Food Sharing across Borders: First Observation of Intercommunity Meat Sharing by Bonobos at LuiKotale, DRC

**DOI:** 10.1007/s12110-018-9311-9

**Published:** 2018-04-05

**Authors:** Barbara Fruth, Gottfried Hohmann

**Affiliations:** 1 Faculty of Science, School of Natural Sciences and Psychology, Liverpool John Moores University, Liverpool, L3 3AF UK; 2 Centre for Research and Conservation, Royal Zoological Society of Antwerp, Koningin Astridplein 20-26, B-2018 Antwerp, Belgium; 3 Department of Primatology, Max-Planck-Institute for Evolutionary Anthropology, Deutscher Platz 6, D-04103 Leipzig, Germany

**Keywords:** Food sharing, Intercommunity encounters, Bonobo, *Pan paniscus*, Human evolution

## Abstract

Evolutionary models consider hunting and food sharing to be milestones that paved the way from primate to human societies. Because fossil evidence is scarce, hominoid primates serve as referential models to assess our common ancestors’ capacity in terms of communal use of resources, food sharing, and other forms of cooperation. Whereas chimpanzees form male-male bonds exhibiting resource-defense polygyny with intolerance and aggression toward nonresidents, bonobos form male-female and female-female bonds resulting in relaxed relations with neighboring groups. Here we report the first known case of meat sharing between members of two bonobo communities, revealing a new dimension of social tolerance in this species. This observation testifies to the behavioral plasticity that exists in the two *Pan* species and contributes to scenarios concerning the traits of the last common ancestor of *Pan* and *Homo*. It also contributes to the discussion of physiological triggers of in-group/out-group behavior and allows reconsideration of the emergence of social norms in prehuman societies.

Cooperative behaviors such as hunting and food sharing play an important role in constructing models of human origins (Dart 1953; Gurven and Hill 2009; Isaac 1978; Jaeggi and Gurven 2013; Lovejoy 1981; Stanford and Bunn 2001). Scrutiny of these behavioral patterns in our closest living relatives, the chimpanzee (*Pan troglodytes*) and the bonobo (*Pan paniscus*), provides potential insights into how our last common ancestor may have acted when dividing food with others (Marchant 2004).

More than 50 years ago Goodall (1963) recognized the importance of meat and meat sharing in chimpanzee society. Hunting and meat eating became a lens for interpreting male chimpanzee politics (Nishida et al. 1992), with sharing seen as a strategy by males to secure allies (Mitani 2009; Nishida et al. 1992; Nishida and Hosaka 1996) as well as reproductive partners (Gilby et al. 2007; Gomes and Boesch 2009; Nishida and Hosaka 1996; Stanford 1998; Tutin 1979; Watts and Mitani 2002). Only recently, Gilby et al. (2010) published substantial evidence for meat sharing in chimpanzees not being sexually motivated. For a long time, hunting in bonobos was considered to be rare (Badrian and Malenky 1984; Ihobe 1992; Kano and Mulavwa 1984) and division of food among adults was thought to be restricted to plant foods (Badrian and Badrian 1984; Kano 1980; Kuroda 1984). More recent data revealed a number of novel traits. First, at least in some populations, bonobos hunt primates and other vertebrates and share meat with each other (Fig. 1; Fruth and Hohmann 2002; Hohmann and Fruth 2008; Surbeck et al. 2009). Second, independent of the nature of the food item, whether meat or large fruits, food sharing is often controlled by adult females (Fruth and Hohmann 2002; Yamamoto 2015; for an exception, see Fig. 2). Third, independent of item size (meat and large fruit vs. piths and leaves) and item acquisition (solo or as group), food begging and food transfers occur (Fig. 3) and may reinforce social ties (Goldstone et al. 2016; Yamamoto 2015). The new data from wild bonobos not only shed light on the behavioral diversity within and across the genus *Pan* but importantly offered new perspectives on the role of food sharing in human evolution (Fruth 1998; Fruth and Hohmann 2002; Hohmann and Fruth 2008; Ihobe 1992; Surbeck et al. 2009). In contrast to the findings for chimpanzees, the substantial amount of plant food sharing documented for bonobos suggests a reconsideration of the historical claim that sharing in hominins evolved solely as a consequence of the hunting and subsequent sharing of meat (Fruth and Hohmann 2002). Instead, it may also be important to consider package size, whether particular foods can be monopolized, and the underlying social relationships between the females involved. Thus, rather than focusing solely on energetic gains as pacemakers for hominin politics, it may be at least as useful to analyze the processes by which highly contested and monopolizable foods are acquired and shared (Fruth 1998; Fruth and Hohmann 2002). In particular, such processes may occur independently of size and energetic value of the foods in question (Goldstone et al. 2016).

**Fig. 1:**
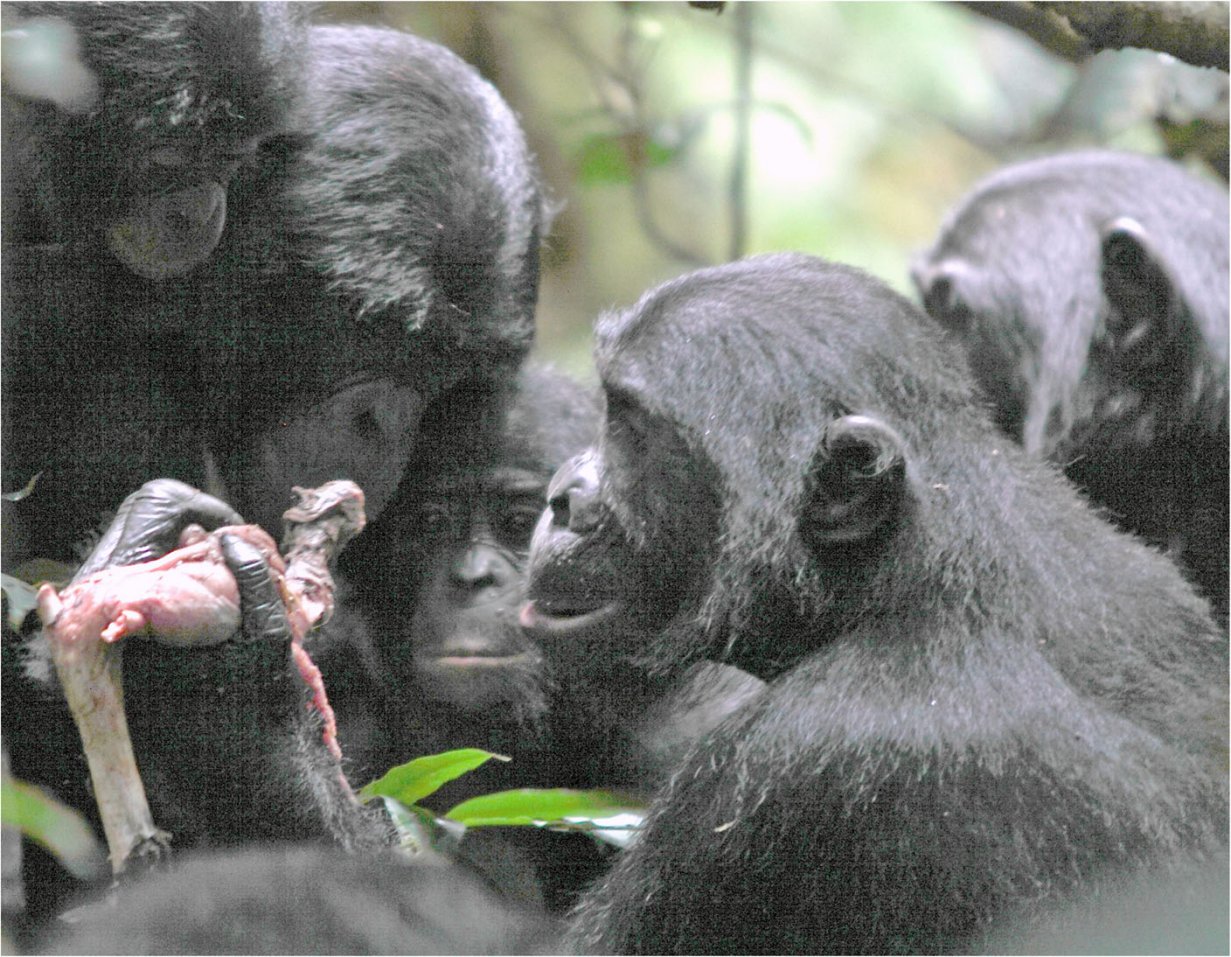
Peering for a piece of meat, bonobos gather around the owner of a duiker’s leg (LuiKotale Bonobo Project /Robin Loveridge)

**Fig. 2:**
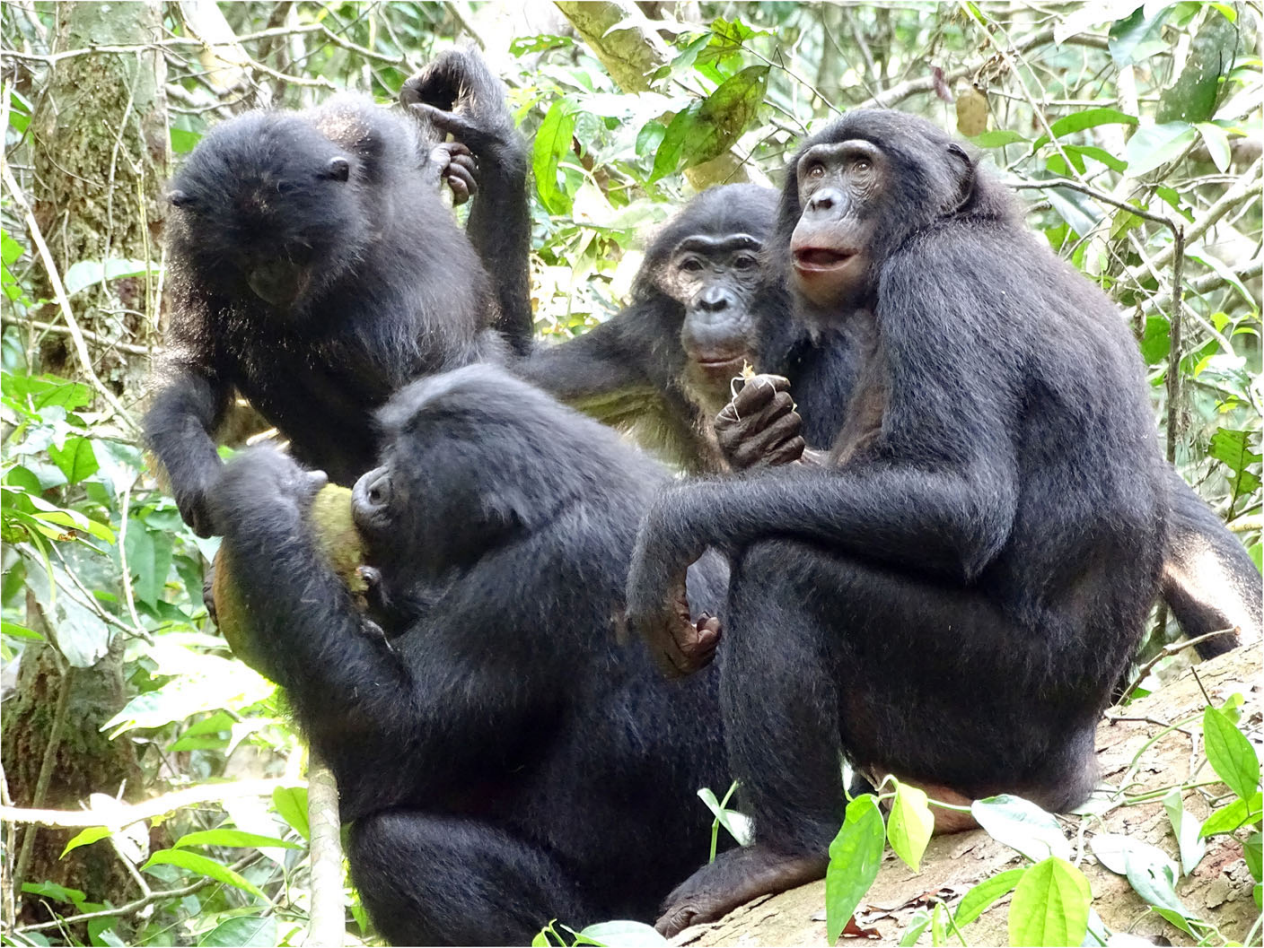
African breadfruit (*Treculia africana*) sharing: a party is gathered around the owner (in this case, a male). (LuiKotale Bonobo Project/Barbara Fruth)

**Fig. 3:**
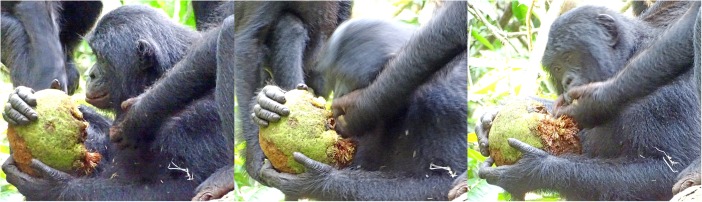
The owner of an African breadfruit rips off a piece with his teeth and transfers it into the open palm of the beggar (LuiKotale Bonobo Project/Barbara Fruth)

In chimpanzees, food-sharing has come into play as a releaser for oxytocin, playing a key role in individual bonding and long-term cooperation (Wittig et al. 2014). In orangutans (*Pongo pygmaeus*) and bonobos, the evidence suggests that solicitors are testing the tolerance of and support from higher-ranking possessors, independent of the food’s nutritional value (Goldstone et al. 2016; van Noordwijk and van Schaik 2009). This aligns well with bonobos being known for high levels of tolerance (Hare et al. 2007) and moderate levels of aggression (Wilson et al. 2014) between individuals. Although these studies address social relations within ape groups or communities, little is known about the importance of food sharing between groups or communities.

Most diurnal primate taxa live in groups, and group membership is relatively stable. In some species, spatial associations are flexible, with individuals forming aggregations that vary in size and composition. This *fission-fusion* social organization affects social interactions among members of the same community and may be used to gauge social relationships among residents (Aureli et al. 2008). The assignment of individuals to a given community is usually based on their use of space. Members of the same community travel together, have overlapping mobility patterns, and associate with each other. Individuals of different communities are spatially separated and associate rarely or never. The nature of social interactions between individuals of different groups may vary with group size (Benadi et al. 2008; Wilson et al. 2012), location of encounter (Crofoot et al. 2008), and resources (Boinski et al. 2002; van Schaik 1999).

In bonobos, relations between neighboring communities are relaxed and peaceful when compared with chimpanzees (Fruth et al. 1999; Furuichi 2011). Until this case study, observations of direct encounters at LuiKotale ranged from 1 to 3 per year, with interactions between members of different communities ranging from affiliative to aggressive. Although aggressive displays are a common feature of encounters, physical aggression and injuries are less frequent. At Lomako, they were witnessed in one third of all encounters (Hohmann and Fruth 2002). The low rates of aggression contrast with what is known from chimpanzees and emphasize the fact that bonobos tend to solve conflicts by nonaggressive behaviors (de Waal 1989). This tolerance toward nonresidents has been explained by resource distribution and abundance, and by female dominance (Furuichi 2011). This raises the question of whether this tolerance disappears when resources can be monopolized. Here we report the first case of meat sharing observed during an intercommunity encounter, in which one individual in possession of an adult forest antelope (*Cephalophus callipygus*) shared with members of its own group as well as with members of the neighboring community.

## Material and Methods

**Fig. 4:**
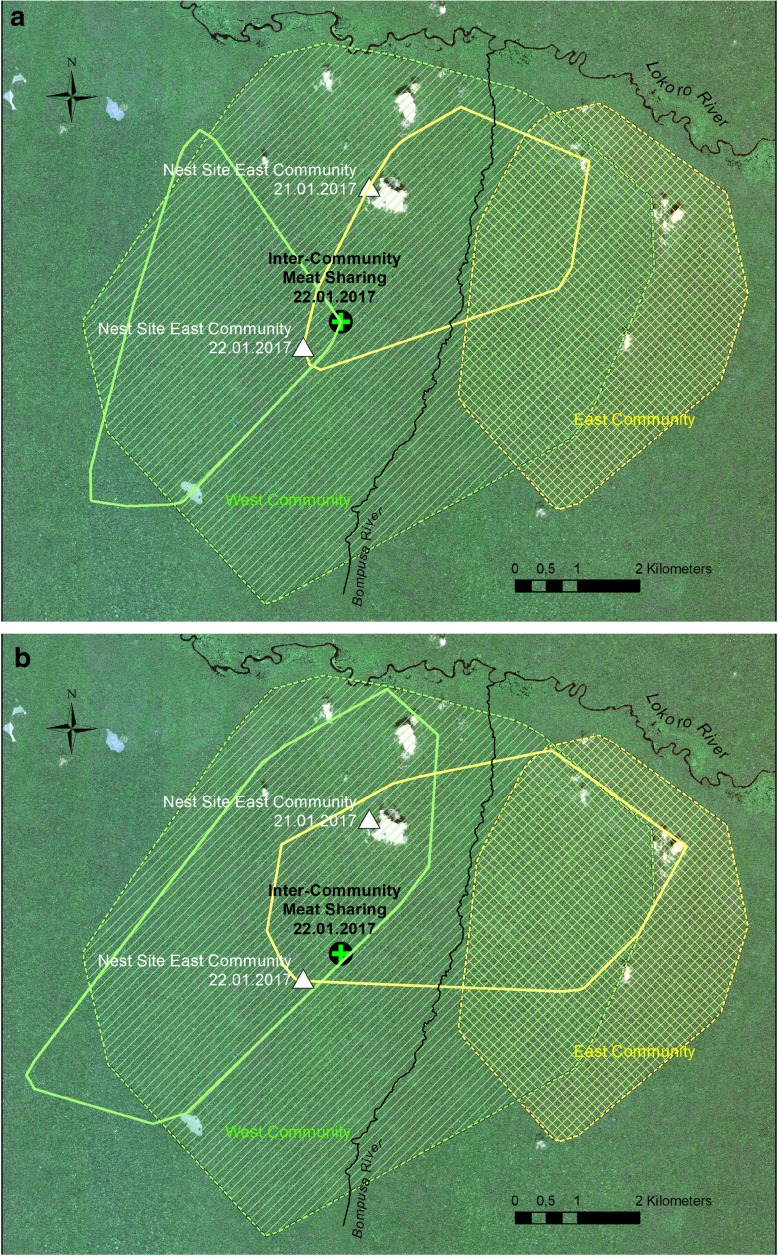
Home ranges of W (simple hatched lines) and E (cross-hatched lines) communities. Subranges show the areas covered during the 22 days **a** prior to the sharing event (between January 1 and 22, 2017) and **b** after the sharing event (between January 22 and February 13, 2017)

Data are from two habituated communities of bonobos, the Bompusa West (W) and East (E) communities at LuiKotale, Democratic Republic of the Congo (Hohmann and Fruth 2003). Table 1 provides details of community size and composition. The core ranges of the two communities are on either side of the Bompusa River (Fig. 4), a small stream draining south to north into the Lokoro, a large river setting the northern limit to both ranges. Members of the W community are fully habituated since 2007; those of the E community, since 2015. All bonobos are known individually. Kinship of residents of the W community has been assessed with molecular markers (Surbeck et al. 2017). Behavioral (focal and ad libitum observations) and ranging data (GPS track logs) are collected daily by research assistants. Track logs are stored in Garmin BaseCamp V4.6.2, cleaned, and depicted as home ranges in ArcGIS V10.2.2 using the Minimum Convex Polygon Tool. Food sharing is defined as unrestricted food transfer from one individual to another (Feistner and McGrew 1989), differing from co-feeding in that one individual is in control of the food item. Based on records from both communities collected at LuiKotale between May 2016 and April 2017, hunting and/or meat consumption occurred on average twice per month. At 59%, duikers were the most frequent prey species of all hunts (*n* = 22). Independent of prey species, most hunts appear to be opportunistic (unpublished data).

**Table 1.** Age and sex composition of the West (W) and East (E) communities

		Adults & adolescents	Juveniles & infants	Adults soliciting	Adults obtaining
West Community					
Community^*a*^	F	18	9		
	M	8	4		
Food-Sharing Party^*b*^	F	11	9	6	5
	M	3	4	0	1
East Community					
Community^*a*^	F	12	5		
	M	7	3		
Food-Sharing Party^*b*^	F	10	5	4	1
	M	5	3	0	0

^*a*^All individuals of the community

^*b*^Individuals present or involved in the intercommunity food-sharing event of January 22, 2017

## Report

On January 21, 2017, subgroups of both communities, known as *parties*, were followed by different teams of observers on either site of the Bompusa River until they stopped at night to construct their nest. On January 22, a mixed party of bonobos from the E community with 10 mature females and 5 mature males (immatures not counted) and from the W community with 12 mature females and 3 mature males (immatures not counted) was contacted around 12:00 h in the forest west of the Bompusa River. At 14:04 h Camillo, the alpha male of the W community, caught a duiker (*Cephalophus callipygus*) and was immediately approached by individuals from both communities. At 14:11 h Camillo moved into the crown of a tall tree where he was followed by nine females and their offspring. During the following 30 min, five adult females from the W community (Gwen, Iris, Uma, Rio, Katie) and four females from the E community (Evimba, Agbaya, Kimya, Lombe) solicited parts of the duiker being in Camillo’s possession. Solicitation involved behaviors such as peering and stretched out hands but no aggression or forceful taking. As in other cases, the transfer of food from the male to females was passive. All five females from the W and one female from the E community (Agbaya) obtained small pieces from Camillo that were immediately ingested. Then, Agbaya (E community) removed the duiker’s entire head (14:36 h) and shared meat from this piece with her offspring and with adult females from both communities. Iris (W community) obtained a leg from the carcass held by Camillo and shared this with her offspring and with adult females from her community. Behavioral records taken during this time included multiple genito-genital contacts (GG-rubbing) between adult females of the two communities as well as between co-resident females. One copulation between a male from the E community and a female of the W community was witnessed, as was grooming between one female from the E community and one female from the W community. At 15:02 h the primary possession of the carcass went from Camillo to an unidentified adult female. Observation was obstructed by foliage and heavy rain and became even more difficult when bonobos involved in food sharing climbed higher into the canopy. At 15:02 h, observations ceased when the bonobos moved to a position that was not visible to the human observers. Members of the W community were not relocated, and members of the E community were encountered later at their nest site, where the first individual built its nest at 16:38 h, the last at 17:11 h. Despite the presence of seven other males, two from the W community and five from the E community, no male other than Camillo participated in the interactions during the meat-sharing episode. No aggression was observed among females, between males and females, or among males, a behavior not uncommon during other community encounters (unpublished data).

In order to assess the spatial use of the respective home ranges in the context of this sharing event, track logs from January 1 to 22, 2017 (Fig. 4a), and the equivalent days after the sharing event (January 23 to February 13, 2017; Fig. 4b) were plotted for each community and set into context of the year-round home ranges of each community in 2016.

Community home ranges for 2016 are represented by minimum convex polygons, with individuals in the E community ranging east of the Bompusa River (cross-hatched area). Individuals in the W community occupied a larger area but had ranged for several months in the far southwestern area of their range (simple hatched area). By mid-January, W individuals had returned to the more central area of their range, whereas E individuals had considerably intruded into what could be considered the core area of the W community’s home range. Despite the relaxed encounter, continued observation of ranging patterns after the meat-sharing episode shows the E community returning to their central range, rather than continuing their presence within the W community’s home range.

## Discussion

Social life in humans is said to be regulated by social norms, such as food sharing, cooperative hunting, or warfare (Kaplan and Gurven 2005; Wrangham and Glowacki 2012). These norms are meant to constrain selfish behavior and reinforce altruism. Because they act on a group level, they are likely to support preferences for the members of one’s own social group. These social norms become evident in intergroup conflicts, with in-group members benefitting, while out-group members don’t. The term “group” in human societies varies from a family, as the smallest unit, to various levels of communities, linguistic entities, ethnically or ideologically homogenous units—all of which are a matter of definition. There is plenty of evidence of intragroup food sharing in humans (Kaplan and Gurven 2005), but sharing with non-group members is less frequent, although present in traditional societies such as the! Kung (Lee 1979) or the Eipo (Schiefenhövel 2014). Sharing with strangers is often seen as a unique attribute, setting humans apart from other primates (Boyd and Silk 2000). In experimental settings, captive orangutans and bonobos also share food with members of other groups (Kopp and Liebal 2016; Tan et al. 2017; Tan and Hare 2013). In experiments, Tan and Hare (2013) have demonstrated that bonobo food sharing is prosocial and a way to facilitate contact with strangers. Interestingly, however, when given the choice to eat food alone or to let another bonobo into a confined space to eat together, bonobos preferred to eat alone and not to share with a groupmate. This was true for familiar individuals in Leipzig Zoo (Bullinger et al. 2013) as well as individuals said to be less familiar in Lola ya bonobo (Tan and Hare 2013), providing no support for voluntary co-feeding or food sharing. Both *Pan* species in these experiments preferred company in the absence (rather than in the presence) of food. To what extent apes living in the same facility are “strangers” is a matter of definition, but spatial proximity, and acoustic and visual contact, are likely to create familiarity that exceeds that of neighboring communities in the wild. However, it is noteworthy that both studies found that the motivation to share with others varied with familiarity, with owners of food preferring to share with less-familiar individuals rather than with group mates (Bullinger et al. 2013; Tan and Hare 2013).

Since 2015, the rate of encounters between members of the E and W community at LuiKotale was low (twice per year), but beginning in early 2017, members of both communities encountered each other more frequently (on average once per month; unpublished data). Thus, until recently, residents from both communities neither heard nor saw each other for long periods of time; nonetheless, because bonobos are characterized by female dispersal, social ties may exist from females having transferred prior to 2007.

This observed familiarity across communities is yet another example of bonobo social tolerance, whereas it is unthinkable among chimpanzees. There, intergroup relationships are typically violent, with males being highly intolerant toward members of other communities, resulting in severe and often lethal aggression against individuals of both sexes (Wilson et al. 2014). This seems to be independent of food resource characteristics (clumped vs. dispersed; abundant vs. scarce) since it occurs across different field sites and seasons. Chimpanzees serve as a prime example of resource-defense polygyny (Manson and Wrangham 1991; Williams et al. 2004), whereas in bonobos, neither home ranges nor females are defended as much (Fruth et al. 1999; Furuichi 2011). Nevertheless, home ranges are pretty stable over time and physical aggression is known from initial stages of intercommunity encounters, although they may diminish as the encounter continues, leading to grooming, sex, and co-feeding.

The episode of meat sharing between members of two different bonobo communities reported here is noteworthy in several respects. The possession of a forest antelope by an adult male and the sharing of its meat with females from another community reveals a new dimension of social tolerance in wild bonobos. Although participation in sharing is part of a young immigrant female’s integration process, here, the possessor shared meat with an older multiparous female who was a long-term resident of the neighboring community. Females who obtained meat shared with females from their own as well as from the other community. None of these transfers involved aggressive behavior, and transfer of meat was induced by peering, begging gestures, or simply by waiting, indicating an unusual level of social tolerance previously not observed.

What are potential explanations for this new dimension of social tolerance? First, we cannot exclude at this point that the females involved in intercommunity food sharing are relatives. Ongoing analyses of the genealogy of the East community will enable us to identify kin relationships between adult members of the two communities, but we do have evidence that cooperation between unrelated females may override genetic ties (Parish and de Waal 2000) and lead to a social network based on reciprocity and mutuality rather than kinship (Moscovice et al. 2017). Second, the social selection hypothesis, which postulates the occurrence of competition for being chosen as either a social partner or a mate (West-Eberhard 1983), could also explain tolerant sharing with unfamiliar individuals. This hypothesis finds support in the bonobos’ concealed ovulation, making mate guarding and male-male competition less effective than in other species, and giving way to female choice (Douglas et al. 2016; Furuichi et al. 2012; Reichert et al. 2002). If tolerance is a trait that females select in the context of mate choice, a male displaying tolerance across community boundaries might enhance his reproductive success. However, this hypothesis has been challenged by data from different captive settings, in which bonobos have demonstrated pronounced despotic behavior (Jaeggi et al. 2010), and a detailed review of long-term data from two wild chimpanzee communities refuting the meat-for-sex hypothesis (Gilby et al. 2010). Third, in terms of the neuroendocrine physiology of human and primate cooperation in general, and the role of oxytocin for in-group bonding in particular (Bernhard et al. 2006; Trumble et al. 2015; Wittig et al. 2014), parochial altruism is challenged by our observation, since extending sociopositive interactions to the intergroup level may serve as an alternative for group aggression, providing similar physiological rewards.

In the future we need to identify the initiators of explorations into neighboring home ranges. Is it males or females who make the initial forays, and what are the potential benefits for each sex? What are the benefits that males may derive from sharing with females other than those who reside in their community? Combining paternity data with observations of interactions between males and females during intercommunity encounters can be used to address these and related questions.

This observation provides yet another glimpse into the behavioral divergence observed in both *Pan* species. It reminds us of the enormous variability we find in these sister species, wherein the use of normative conformities ranges from xenophobic behavior performed to maintain community boundaries to xenophilic behavior allowing peaceful relationships beyond those boundaries. One behavioral pattern serving as a starting point for the evolution of societal levels is the genus *Pan*’s “protolanguage” (Moffett 2013), reflected in specific elements of their distance vocalization coding group identity (Crockford et al. 2004). With this in mind, Moffett (2013) has timed emergence of social identity beyond individual recognition after the *Pan-Homo* split. Food sharing in bonobos contains societal labels typically found in humans (sensu Moffett 2013), such as food preferences (what to share), values, and rituals (how to beg, negotiate, and share). It thus represents a shared identity that enables creation of familiarity independent of individual recognition, allowing not only intergroup trust but strangers to be treated as friends.
